# Import of antibiotics by air travellers – an observational study at airports in Sweden and Norway

**DOI:** 10.1186/s13756-026-01816-6

**Published:** 2026-09-11

**Authors:** Cecilia Lenander, Amelie Persson, Hajer Al Hassani, Katarina Hedin, Sigurd Høye, Siri Jensen, Pär-Daniel Sundvall, Sofia Sundvall, Anna Moberg

**Affiliations:** 1 Center for Primary Health Care Research, Department of Clinical Sciences Malmö, Lund University, Malmö, Sweden; 2 Department of Clinical Sciences in Malmö, Family Medicine, Lund University, Malmö, Sweden; 3 Department of Strategic Healthcare Development and Safety, Skåne University Hospital, Malmö, Sweden; 4 Department of Pharmacy, Uppsala University, Uppsala, Sweden; 5 Department of Health, Medicine and Caring Sciences, Linköping University, Linköping, Sweden; 6 Futurum, Region Jönköping County, Jönköping, Sweden; 7 The Antibiotic Centre for Primary Care, Department of General Practice, University of Oslo, Oslo, Norway; 8 General Practice/Family Medicine, School of Public Health and Community Medicine, Institute of Medicine, Sahlgrenska Academy, University of Gothenburg, Gothenburg, Sweden; 9 Research, Education, Development & Innovation, Primary Health Care, Region Västra Götaland, Gothenburg, Sweden; 10 Primary Healthcare Center Kärna, Region Östergötland, Linköping, Sweden

**Keywords:** Antibiotics, Antimicrobial resistance, Imports, Air travel, Customs

## Abstract

**Background:**

Overuse and misuse of antibiotics are two of the main drivers of antimicrobial resistance (AMR). In some countries antibiotics can be purchased over the counter, whereas in Scandinavia and most of Europe, antibiotics require a prescription. It is permitted to bring medicines for personal use to Scandinavia. This raises concerns about whether antibiotics are being imported into low-prescribing countries, such as Scandinavia, via international airports. We aimed to investigate the quantity and types of antibiotics imported by air travellers entering Norway and Sweden, based on routine customs inspections at international airports.

**Methods:**

A cross-sectional observational study was conducted during daytime over five weekdays at three international airports in Sweden and Norway. Anonymous data including the presence, type, and quantity of antibiotics, were registered alongside routine customs inspections. Defined daily doses (DDDs) were calculated for oral antibiotics. Substances were categorised according to the World Health Organization’s (WHO) AWaRe classification (Access, Watch and Reserve).

**Results:**

Out of around 137,500 travellers arriving from abroad, 406 (0.30%) were inspected at routine customs inspections, of whom 57 (14%) carried antibiotics, corresponding to 643 DDDs. Inspected travellers from outside the EEA more often carried antibiotics than those within EEA (*p* < 0.001). More than one-third of the antibiotics were classified as ‘Watch’ according to the AWaRe classification.

**Conclusion:**

We found that 14% of inspected international air travellers carried antibiotics. These antibiotics may be used without medical guidance, raising health risks and the risk of AMR. The findings highlight a potential need for public education and/or strengthened regulatory measures.

## Background

Overuse and misuse of antibiotics are two of the main drivers behind the development of antimicrobial resistance AMR [1]. The World Health Organization (WHO) highlights AMR surveillance and the use of antimicrobial agents as important priorities for tackling AMR, which requires efforts across multiple sectors, including food production, animal health, and the environment [2].

To improve antibiotic stewardship and ensure appropriate use globally, WHO has developed the AWaRe classification (Access, Watch, Reserve). This system categorises antibiotics based on their risk of promoting antimicrobial resistance and their importance in medical treatment [3].

In Scandinavia and most other European countries antibiotics require a prescription, whereas in some countries they are available over the counter [4, 5]. According to the Eurobarometer 2022, 8% of Europeans report using antibiotics without a prescription [6] and this has remained unchanged over the past 20 years [7].

Since antibiotic resistance threatens the effectiveness of life-saving treatments, every dose matters. In Scandinavia there is a long tradition of strategic work to preserve the effectiveness of antibiotics in humans and animals [8, 9]. An important part of the work against AMR is surveillance of antibiotic prescriptions, requiring national registers, which are available in Scandinavia [10, 11]. However, prescriptions might not correspond to consumption. The national strategy on antibiotic resistance in Sweden and Norway elucidates the importance of guiding the public on safe use of antibiotics, and the Swedish strategy advises the public against bringing antibiotics from abroad [12, 13]. However, travellers are allowed to bring medicines, including antibiotics, for personal use. Within the European Economic Area (EEA), up to a one-year supply is permitted, while from outside the EEA a three-month supply of non-controlled medicines is permitted [14].

Customs officials at airports routinely conduct inspections of selected travellers to ensure compliance with regulations regarding prohibited items. The selection is based on tips from law enforcement and assessments made by customs officers. The entry of antibiotics through international airports remains an under-researched topic.

### Aim

This study aimed to investigate the quantity, types and classification of antibiotics carried by air travellers entering Norway and Sweden, based on routine customs inspections at three major international airports.

## Methods

This was a cross-sectional observational study. The study population consisted of international travellers entering Stockholm Arlanda Airport and Gothenburg Landvetter Airport in Sweden, and Oslo Gardermoen Airport in Norway. Approximately 20 million international travellers arrive to these three airports annually.

Data were collected over five weekdays at each airport (13–17 October 2025 at Arlanda, and 20–24 October 2025 at Landvetter and Gardermoen) during routine customs inspections carried out by Swedish and Norwegian Customs officials. Airport authorities provided data on the number of arriving travellers during the inspection period.

Observations were conducted alongside routine customs procedures, Monday through Friday between 7 am and 4 pm. Research personnel had an administrative role and documented the presence of antibiotics detected during the inspections. Travellers were selected for inspection by customs officers based on routine risk assessment and operational priorities. At Arlanda and Gardermoen, selected travellers’ luggage was initially screened using X-ray and only opened if the scan revealed items requiring further examination. A customs inspection was therefore only recorded when the luggage was physically opened. In contrast, at Landvetter, all luggage belonging to travellers chosen for inspection was routinely opened. Swedish customs may inspect EEA travellers only on suspicion of illegal import of any kind, but travellers from other parts of the world may be subject to random inspections. Norway is part of the EEA, but not the EU customs union, which means that all travellers to Norway may be subjected to random inspection.

During each inspection, information including initial country of departure and whether any antibiotics were identified was registered. If antibiotics were detected, additional information on substance, strength, pharmaceutical form, and quantity was recorded. All data were anonymous. When the medicine was unclear or text was written in a non-Latin alphabet, the packet was photographed to enable identification, and translation tools were used when needed (Google Translate).

At the end of each day, customs officers were asked whether the day was representative regarding workload, number of inspections, and seizures, and if not, how it differed.

### Data management and statistics

Data are presented descriptively as numbers, proportions, and defined daily doses (DDD) calculated for oral antibiotics [15]. Antibiotics were classified according to ATC codes and thereafter categorised according to the AWaRe classification of antibiotics [3]. The total quantities of imported antibiotics were summarised according to the country of departure. Countries spanning two continents were classified based on where the majority of their territory lay. Group comparisons between country of origin and type of antibiotic were performed using chi-square tests. Odds ratios (ORs) with 95% confidence intervals (CIs) were calculated using Mantel-Haenszel equation. Statistical significance was set at *p* < 0.05.

## Results

In total, 137,519 international air travellers arrived at the airports during the study period, of whom 406 (0.30%) were inspected (Table 1). Antibiotics were found in 57 (14%) of the inspected travellers, corresponding to 643 DDD and 11.3 DDD per traveller detected with antibiotics. The proportion of travellers carrying antibiotics was higher at Arlanda (23%) than at Landvetter and Gardermoen (10% at both airports) (Table 1).

**Table 1:** Number of arriving and inspected international travellers at each study airport and in total

Airport	Arriving international travellers n	Inspected travellers n	Proportion of travellers inspected, % (95%CI)	Travellers with antibiotics n	Proportion with antibiotics among inspected travellers, % (95%CI)
*ARLANDA*	65,035	117	0.18 (0.15–0.22)	27	23 (16–32)
*LANDVETTER*	17,484	221	1.3(1.1–1.4)	23	10 (7–15)
*GARDERMOEN*	55,000*	68	0.1*	7	10 (5–20)
*TOTAL*	**137,519**	**406**	**0.30 (0.27–0.33)**	**57**	**14** (11–17)

The proportion of inspected travellers relative to total arrivals is presented

*Approximation based on international arrivals from the whole week

**Fig. 1:**
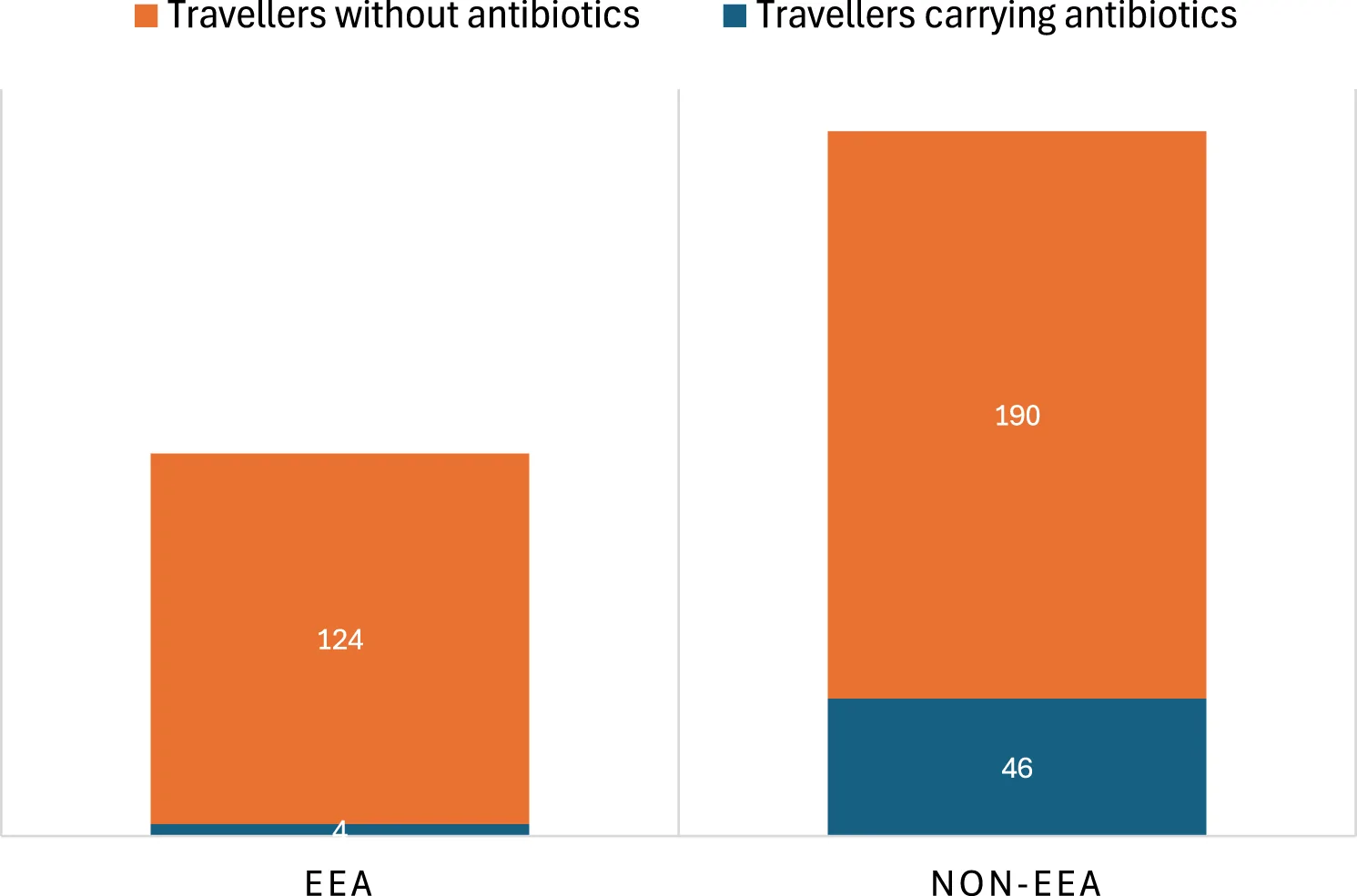
Number of inspected air travellers arriving from the European Economic Area (EEA) and from non-EEA countries, with and without antibiotics detected during routine customs inspections at Arlanda, Landvetter and Gardermoen airports during five weekdays in October 2025. The y-axis shows the number of travellers. Data from two days at Arlanda were excluded due to missing information regarding country of departure

Antibiotics were found in 19% of travellers arriving from outside the EEA, compared with 3% within EEA, corresponding to an odds ratio of 8.6 [95% CI 3.4–22; *p* < 0.001]. Travellers arriving from outside the EEA were inspected more frequently (Fig. 1). Asia, including the Middle East, exhibited both the highest proportion and the largest number of travellers carrying antibiotics (Table 2). No travellers from South America or Oceania were found to carry antibiotics, which were also the continents with the lowest numbers of inspections.

**Table 2:** Antibiotics detected during routine customs inspections

Continent*	Inspected travellers (n)	Travellers carrying antibiotics (n)	Proportion of travellers carrying antibiotics % (95% CI)
*Africa*	46	8	17 (9.1–31)
Asia**	103	28	27 (20–37)
*Europe*	194	11	5.7 (3.2–9.9)
*North America*	15	3	20 (7.0–45)
*South America*	5	0	0 (0–44)
*Oceania*	1	0	0 (0–79)

The inspections took place at Arlanda, Landvetter, and Gardermoen airports for five days. Inspected travellers are stratified by continent of departure

*Data from two days at Stockholm Arlanda Airport were excluded due to incomplete information on country of departure

**Asia includes the Middle East

### Types of antibiotics

**Fig. 2:**
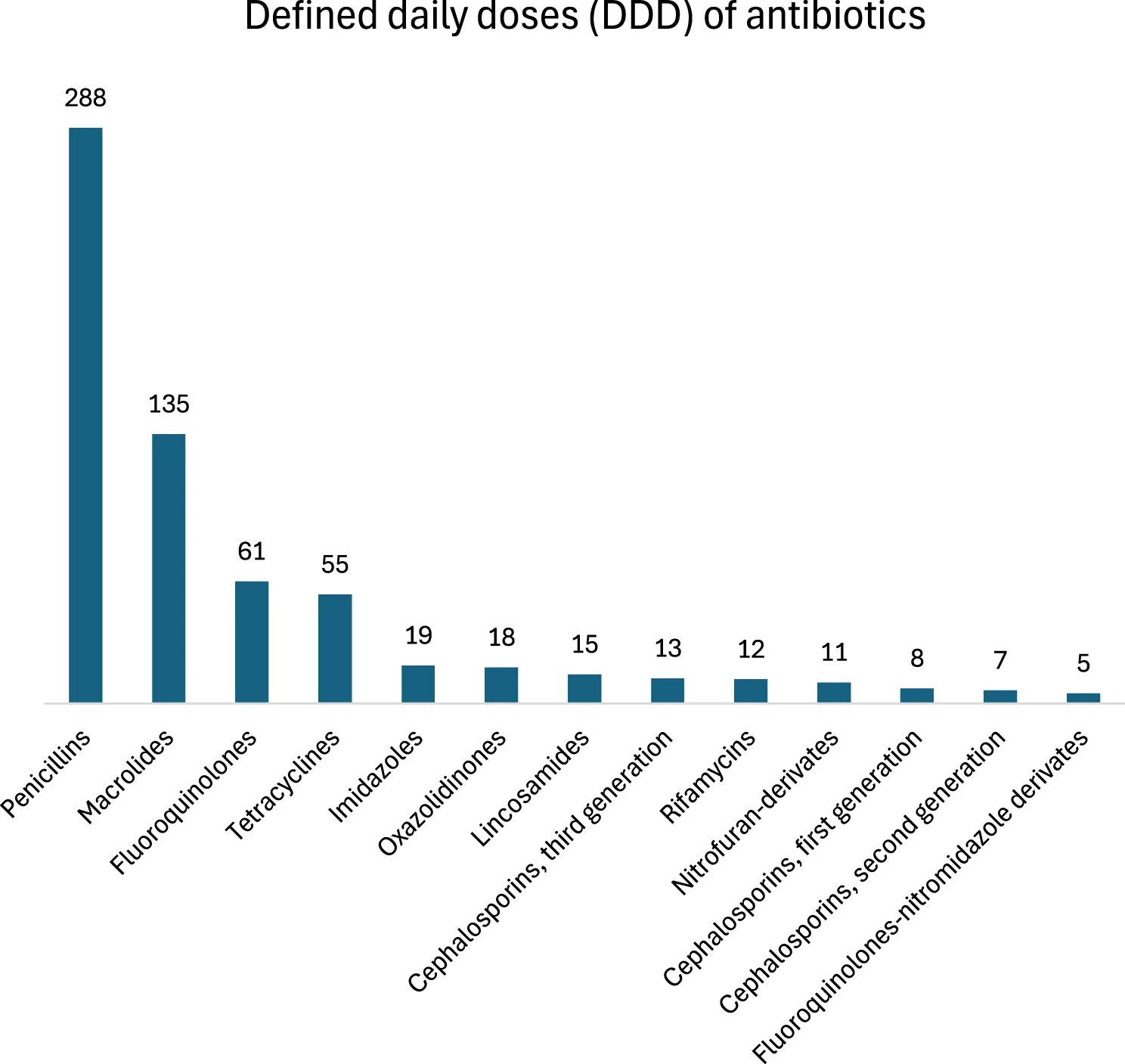
Total defined daily doses (DDDs) of oral antibiotics detected at routine customs inspections during five weekdays in October 2025 at Arlanda, Landvetter and Gardermoen airports grouped by antibiotic class. Furazolidone was excluded due to unavailability of DDD data

In total, 35 antibiotic substances (based on ATC codes) were identified during the customs inspections. Twenty-three substances were in tablet form and could be quantified by the number of tablets, and all but one (furazolidone) could be converted to DDDs (Table 3). A total of 1,700 tablets were detected, corresponding to 643 DDDs, excluding furazolidone for which DDD data were unavailable. Overall, 45% of the DDDs were penicillins, followed by macrolides (21%) (Fig. 2). Oral formulations (tablets, capsules, or oral suspension) and topical formulations (ointments/creams, eye or ear drops) were identified.

**Table 3:** Oral antibiotics detected at the study airports

Antibiotic class	Antibiotic substance	Tablets (n)	DDDs	AWaRe classification
Antibacterial beta-lactam antibiotics: penicillins	amoxicillin	454	133	ACCESS
	amoxicillin-clavulanic acid	334	124	ACCESS
	ampicillin	30	8	ACCESS
	phenoxymethylpenicillin	18	3	ACCESS
	flucloxacillin	40	20	ACCESS
Macrolides	azithromycin	94	127	WATCH
	roxithromycin	16	8	WATCH
Cephalosporins, 1st generation	cefalexin	30	8	ACCESS
Cephalosporins, 3rd generation	cefixime	9	9	WATCH
	cefpodoxime	7	4	WATCH
Cephalosporins, 2nd generation	cefprozil	13	7	WATCH
Fluoroquinolones / nitroimidazole derivatives	ciprofloxacin-ornidazole	10	5	WATCH
	norfloxacin	40	20	WATCH
	levofloxacin	41	41	WATCH
Tetracyclines	doxycycline	89	53	ACCESS
	tetracycline	15	2	ACCESS
Nitrofuran derivatives	furazolidone	220	Data missing	Not applicable
	nifuroxazide	9	3	Not applicable
	nitrofurantoin	30	8	ACCESS
Lincosamides	clindamycin	29	15	ACCESS
Oxazolidinones	linezolid	36	18	RESERVE
Imidazole derivatives	metronidazole	90	19	ACCESS
Rifamycins	rifaximin	36	12	WATCH
	**Total**	**1700**	**643**	

The inspections took place at Arlanda, Landvetter, and Gardermoen airports over five days. Antibiotic class, substance, quantity (number of tablets), calculated DDDs, and Access, Watch and Reserve (AWaRe) classification are shown

The five most common oral substances were amoxicillin, amoxicillin-clavulanic acid, azithromycin, doxycycline, and levofloxacin (Table 3). In total, 27 packages of topical agents were found, among these, fusidic acid was the most common substance, representing 26%.

### AWaRe classification

Of the 35 antibiotic substances detected, 21 (60%) were included in the WHO AWaRe classification and categorised accordingly. Eleven substances were classified as Access, nine as Watch, and one as Reserve. In addition, furazolidone, an antibiotic not authorized for sale or clinical use in Scandinavia, was detected (Table 3). However, furazolidone is not subject to a general prohibition on personal possession or use in humans. Thus, a traveller may potentially bring legally obtained furazolidone for personal medical use. The distribution DDDs according to AWaRe was 61%, 36% and 2.8% respectively.

## Discussion

### Main findings

In this cross-sectional observational study, we detected a substantial amount of antibiotics corresponding to 643 DDDs during routine customs inspections of air travellers during daytime over five weekdays in Sweden and Norway. We also found that air travellers from outside the EEA more often brought antibiotics. Asia, including the Middle East, was the most common region among travellers carrying antibiotics, with antibiotics detected in over one in four inspected air travellers. This may reflect differences in access to antibiotics and patterns of self-medication across regions. Moreover, almost half of the antibiotics found were classified as “Watch” or “Reserve” according to the WHO AWaRe classification. This differs from the distribution of antibiotics prescribed in Sweden and Norway, where 77–86% belong to “Access” [10, 11].

Current regulations permit travellers to bring medications for personal use for either 3 or 12 months, depending on country of departure.

However, it is uncommon for infections to require treatment for three months, and even rarer for twelve months. In such exceptional cases, a prescription is likely to be available. The overall prevalence of such imports remains unknown. Nonetheless, these activities may pose risks beyond contributing to antimicrobial resistance.

### Strengths and limitations

A major strength of this study lies in its observational design, as well as the completion of the registration process with minimal interaction between study personnel and travellers as well as customs officials. Additionally, inspections were conducted as part of routine procedures, meaning our findings represent a minimum estimate of antibiotic importation by air travellers to these countries.

A key limitation is that the population studied was not a random sample; inspections were carried out based on suspicion of illegal imports or other operational priorities. Travellers carrying other illegal drugs or products may also be more likely to carry antibiotics, which could bias the findings. The inclusion period was not considered representative at any of the airports, mainly due to personnel shortages. At Landvetter, two days were affected by an unusually high number of arriving football supporters from Europe, which influenced inspection priorities. The inclusion period was short and may not reflect a typical week, although this likely means our results represent a minimum estimate of antibiotic importation by air travellers. Another limitation is that we did not record whether travellers had valid prescriptions for the antibiotics they carried. Finally, it remains unclear whether the antibiotics were intended for personal use, consumed during travel, or returned to the country of origin.

### Findings in relation to previous studies

Research on antibiotic importation is limited. However, one Norwegian questionnaire-based study found that 3.7% of travellers had purchased antibiotics abroad in the previous year [16]. Our current study observed nearly double that rate among Europeans overall; however, we did not analyse Norwegians separately, so rates could vary among different European nationalities. Additionally, the Norwegian study relied on self-reported data from questionnaires. Data from the 2022 Eurobarometer survey indicated that 11% of Swedish respondents reported using non-prescription antibiotics [6]; however, the report did not indicate where respondents obtained these antibiotics. As our study included travellers of multiple nationalities, this may partly explain the higher proportion observed. Therefore, differences in the observed proportions relative to population-based studies are unsurprising.

In the current study, amoxicillin, with or without clavulanic acid, was the most common substance found, consistent with findings from Operation PANGEA VII in 2014 [17]. However, Operation PANGEA targets drugs sent by mail, not those brought by travellers.

Travellers from certain regions, such as Asia, including the Middle East, were more likely to carry antibiotics. This may reflect differences in access to antibiotics and patterns of self-medication across regions, consistent with a systematic review showing the highest rates of antibiotic self-medication in sub-Saharan Africa and the Middle East [18].

### Implications and further research

Our results show that national numbers on antibiotic sales likely underestimate the national consumption of antibiotics. Antimicrobial stewardship efforts should particularly target individuals who travel frequently to and from high-consumption countries, and educational materials should be tailored and made available in relevant languages. It is important to ensure availability of information regarding the Scandinavian approach to antibiotic use for newly arrived immigrants, since it may differ substantially from practices in their countries of origin.

In this study only 0.3% of international air travellers were inspected, and the extent to which the observed proportion of findings applies to the broader travelling population remains uncertain. If the observed frequency of findings were applied to all international air travellers arriving at these airports (20 million) an illustrative scenario would estimate a theoretical imported volume of approximately 30 million DDDs per year (95% CI: 18–50 million) in Sweden and Norway combined. This estimate is based on 14% of travellers bringing antibiotics with 11.3 DDDs each. Given that the combined population of Sweden and Norway is approximately 16 million, the import corresponds to 2 DDDs per 1,000 inhabitants. In Sweden and Norway, annual antibiotic prescription rates are 11 and 13 DDDs per 1,000 inhabitants, respectively [10, 11]. Thus, the additional volume could correspond to an increase of approximately 15–18%, although this estimate should be interpreted with caution.

Using antibiotics without consultation with healthcare professionals carries risks, such as unnecessary treatment, drug interactions, or overlooked contraindications. Buying antibiotics from unauthorized sources also poses additional risks, including the possibility of falsified medicines. Furthermore, if individuals import antibiotics via air travel for conditions such as upper respiratory tract infections, this may undermine current efforts to reduce AMR in primary care. The findings highlight the need for interventions and public education to reduce the use of non-prescribed antibiotics.

Future research should include qualitative studies as well as studies with wider inclusion, longer timeframes, and data collection outside standard working hours, ideally based on random samples of travellers. To better target educational efforts, future studies should explore cultural differences and perspectives related to antibiotic use.

## Conclusion

Import of antibiotics by air travellers was frequently observed and represent an additional source of antibiotic use in Scandinavia. A substantial proportion of detected antibiotics belonged to the WHO “Watch” category, which could have implications for AMR development and patient safety. These findings support the need for public education and/or strengthened regulatory measures.

## Data Availability

All data presented in the manuscript are available from the corresponding author upon reasonable request.

## Abbreviations

AMR: Antimicrobial resistance
ATC: Anatomical Therapeutic Chemical (classification system)
AWaRe: Access, Watch and Reserve (classification of antibiotics)
CI: Confidence interval
DDD: Defined daily dose(s)
EEA: European Economic Area
EU: European Union
OR: Odds ratio
REK: Regional Committee for Medical and Health Research Ethics (Norway)
WHO: World Health Organization
